# Locating Ships Using Time Reversal and Matrix Pencil Method by Their Underwater Acoustic Signals

**DOI:** 10.3390/s21155065

**Published:** 2021-07-26

**Authors:** Daniel Chaparro-Arce, Sergio Gutierrez, Andres Gallego, Cesar Pedraza, Felix Vega, Carlos Gutierrez

**Affiliations:** 1 Departamento de Ingeniería Eléctrica y Electrónica, Universidad Nacional de Colombia, Bogotá 111321, Colombia; sagutierrezd@unal.edu.co (S.G.); ajgallegog@unal.edu.co (A.G.); capedrazab@unal.edu.co (C.P.); jfvegas@unal.edu.co (F.V.); 2 Directed Energy Research Centre, Technology Innovation Institute, Abu Dhabi P.O. Box 9639, United Arab Emirates; 3 Escuela Naval de Cadetes “Almirante Padilla”, Decanatura Ingenieria Naval, Cartagena 130001, Colombia; cagm.enap@gmail.com

**Keywords:** matrix pencil method, reconstruction, complex natural resonances, poles, data compression, location of boats, time reversal, backpropagation

## Abstract

This paper presents a technique, based on the matrix pencil method (MPM), for the compression of underwater acoustic signals produced by boat engines. The compressed signal, represented by its complex resonance expansion, is intended to be sent over a low-bit-rate wireless communication channel. We demonstrate that the method can provide data compression greater than 60%, ensuring a correlation greater than 93% between the reconstructed and the original signal, at a sampling frequency of 2.2 kHz. Once the signal was reconstituted, a localization process was carried out with the time reversal method (TR) using information from four different sensors in a simulation environment. This process sought to achieve the identification of the position of the ship using only passive sensors, considering two different sensor arrangements.

## 1. Introduction

The singularity expansion method (SEM), a technique developed by Baum in 1996, processes a signal to seek its singularities by analyzing its behavior and principal characteristics, in the time and frequency domain, to finally represent the signal as a function [1]. One of the most studied and proven methods to implement SEM in the time domain is MPM, which calculates the main poles and residuals after a mathematical process using singular value decomposition [2].

MPM has been considered in applications in which, using signals of electromagnetic radiation, it is possible to evaluate the geological formations [3] and to characterize and locate multiple targets according to their shape [4,5]. Correspondingly, it has been recently used in the field of underwater target detection using the information of principal singularities of sonar signals [6], for which an active source is needed.

Considering that the main goal of our project is to calculate the location of a ship in a controlled environment using information from acoustic signals, we opted for using MPM to reduce data. Similarly, we sought to carry out the localization process through TR, a method developed by Fink [7] in 1989. TR calculates the position of a target in an inhomogeneous medium, using information from many sensors validating mathematical properties in the solution of the wave equation. The use of TR ranges from the location of electromagnetic phenomena, such as lighting discharges [8], to the monitoring of structural health problems [9], going by linear networks analysis [10] and in aperture radar imaging [11]. Specifically, on underwater acoustic applications, it is used in the detection of multidimensional signals in aquatic environments [12] and in processing information for UWA communication processes [13,14].

Previous works related to underwater acoustic signals were developed to locate marine fauna, and have also addressed the problem of boats. We found that many of these works have focused on geometric approaches and intelligent passive sensor arrays configuration to efficiently cover large and neuralgic areas [15,16]. Likewise, localization processes have been developed using pattern recognition methods such as game theory and artificial neural networks [17,18,19]. However, this type of approach requires constant feedback from implementation experiences to provide better results. In addition, it is necessary to mention that in our work, we also seek a reduction in information to carry out communication processes through wireless channels. Thus, the processes mentioned in the previous works could compromise the size of the data packages.

Current ship detection methods include a real-time location using GPS and RFID readers [20]. These methods do not detect the vessels without the required components of the system, including irregular ships. This problem has been addressed with methods such as sonar, radar and arrays of passive sensors that receive information from reflected waves coming from an active source [21,22,23]. In recent years, passive location methods include: time of arrival (TOA), direction of arrival (DOA), and Matched field processing (MFP) [24], which in some cases, may have problems due to the presence of non-homogeneous media [25]. We estimate the target’s location using TR on acoustic signals from the ship noise. The process uses passive sensors without resorting to electromagnetic or acoustic radiation in the medium. This reduces the amount of required energy without affecting the environment. In addition, by implementing TR, we can avoid the problems related to the non-homogeneities of the medium.

The method presented here is part of a project for the detection and location of boats using underwater acoustic signals captured by hydrophones. The project intends to record boat signals from buoys located in strategic positions and process this information in a central node positioned on land. The signal on each buoy needs to be sent through a low-speed wireless communication channel. Therefore, the reduction in data is a critical step in this approach. In [26], we introduced this process applied to real signals; however, the considerations to define the optimal number of poles in the reconstruction mean were not taken into account. In this paper, we extend the process by emphasizing the definition of a fixed number of singularities. We discovered that this value depends on the characteristics and nature of the signals to be treated. This way, we state a greater reduction in the data by eliminating the redundant information coming from the complex conjugates.

This paper is composed as follows. Section 2 is about the mathematical process to make MPM and TR, taking into account its principal algorithm and equations. Section 3 talks about the method proposed, including the test of MPM and TR using constructed and natural signals. Section 4 validates the method by the simulation of the location process on signals previously treated with MPM. This, considering different scenarios of the target, sensor array, and others. Finally, Section 5 presents some conclusions and discussion about our process.

## 2. Background

The basic and mathematical theory about the methods of MPM and TR for the application of signals in the time domain is presented below. The purpose of these methods is to define the technical information for their implementation in specific circumstances that are developed in this paper.

### 2.1. Matrix Pencil Method

Matrix pencil method (MPM) is a specific implementation of SEM in the signals collected in the time domain. Through this process, it is possible to create an approximation of the signal based on its principal *M* singularities related to poles ($s_{1}$, $s_{2}$,…, $s_{M}$) and residues ($R_{1}$, $R_{2}$,…, $R_{M}$). These singularities are the complex natural resonances (CNRs) and can define the p−th term of a signal $g_{p}$ of *N* elements as a function to the form:

(1) $$g_{p}=f\left(p\Delta t\right)=f_{p}$$

(2) $$f_{p}=\sum_{i=1}^{M}R_{i}e^{s_{i}p\Delta t},p=1,2,3,\ldots ,N$$

Here, (Δt) is the inverse of the sampling frequency and represents the time between two data of $g_{p}$. Once the CNRs have been found, it is possible to perform a reconstruction of the original signal, the accuracy of which will depend on the value of M (M<N) [2].

Now, (2) can be expressed using the poles ($\gamma_{i}$), considering that ($\gamma_{i}=e^{s_{i}\Delta t}$):

(3) $$f_{p}=\sum_{i=1}^{M}R_{i}\gamma_{i}^{p},p=1,2,3,\ldots ,N$$

Once $f_{p}$ is defined, we can start the MPM process. Firstly, it is necessary to describe the pencil parameter (L). This value defines the relation between the accuracy of CNRs and the computational load. Its denotation is recommended to be a number in the range of ($\frac{N}{3}<L\leq \frac{N}{2}$). Now, we can introduce a *Hankel matrix* (Y) as [27]:

(4) $$Y=\left[\begin{array}{ccccc} f_{0} & f_{1} & \cdot & \cdot & f_{L}\\ f_{1} & f_{2} & \cdot & \cdot & f_{L+1}\\ \cdot & \cdot & \cdot & \cdot & \cdot \\ \cdot & \cdot & \cdot & \cdot & \cdot \\ f_{N-L-1} & f_{N-L} & \cdot & \cdot & f_{N-1} \end{array}\right]$$

By deleting the first and the last row to Y, the matrices $Y_{2}$ and $Y_{1}$ are generated. Thus, taking into account H as the hermitian conjugate, it is possible to determine that the poles $\gamma_{i}$ are the values of λ, hence, the expression (5) represents a singular matrix:

(5) $${\left[Y_{2}\right]}^{H}-\lambda {\left[Y_{1}\right]}^{H}$$

The process in Equation (5) implies a considerable computational charge, which does not allow ut to be solved using traditional methods. Therefore, the problem can be addressed using the *singular value decomposition* (SVD) in the Y matrix:

(6) $$Y=USV^{H}$$

At this point, it is necessary to define the M value. Remember from Equation (2) that this number is related to the fundamental frequencies of the signal. Thus, the accuracy and the computational charge in the reconstruction step are directly proportional to this quantity. Considering the above information, the choice of M is a very important step in the process. Sarkar in [2] defines M based on the significant digits of the division:

(7) $$\frac{\sigma_{c}}{\sigma_{max}}\approx 10^{d}$$

where $\sigma_{c}$ represents the *c*-th singular value in the diagonal of the *S* matrix in Equation (6), and $\sigma_{max}$ is the largest singular value (located in position 1.1). Here, *d* is the number of significant decimals that you should to use in the process [28].

Let us define the $V^{\prime }$ matrix as the first M rows of *V*. Now, it is possible to remove the last and the first columns of $V^{\prime }$ to create the $V_{1}$ and $V_{2}$ matrices, respectively. Now, the problem in Equation (5) is reduced to finding the values of λ such that Equation (8) is singular [27]:

(8) $${\left[V_{2}\right]}^{H}-\lambda {\left[V_{1}\right]}^{H}$$

The problem to find λ values in Equation (8) can be addressed with the equivalence defined by the eigenvalues of the ${\left[V_{1}^{H}\right]}^{+}\left[V_{2}^{H}\right]$ matrix (where “+” represents the *Moore–Penrose pseudo-inverse*). These eigenvalues are equivalents to $\lambda_{i}$ because of the matrix in Equation (8) is singular. In order to find this information, it is necessary to apply the SVD again. Then, by decomposing $V_{1}$, we have:

(9) $$V_{1}=U_{v}S_{v}V_{v}^{H}$$

Thanks to SVD, it is possible to replace ${\left[V_{1}^{H}\right]}^{+}$ with the approximation presented in Equation (10), where $S_{v}^{\prime }$ is a square diagonal matrix defined as $1/(S_{v})$, of the size MXM:

(10) $${\left[V_{1}\right]}^{+}\left[V_{2}^{H}\right]=V_{v}S_{v}^{\prime }U_{v}^{H}\left[V_{2}^{H}\right]$$

The eigenvalues in the problem of Equation (10) can be solved using traditional mathematical methods, so it is possible to calculate the poles $\gamma_{i}$ and its coefficients $s_{i}$ as follows [4]:

(11) $$s_{i}=\frac{1}{\Delta t}log\left(\gamma_{i}\right),i=1,2,3,\ldots ,M$$

After calculating the poles of the function, it is necessary to find the values of the residuals $R_{i}$. For this purpose, a *Vandermonde matrix* was generated in order to solve the next system of equations:

(12) $$\left[\begin{array}{ccccc} 1 & 1 & \cdot & \cdot & 1\\ \gamma_{1} & \gamma_{2} & \cdot & \cdot & \gamma_{M}\\ \cdot & \cdot & \cdot & \cdot & \cdot \\ \cdot & \cdot & \cdot & \cdot & \cdot \\ \gamma_{1}^{N-1} & \gamma_{2}^{N-1} & \cdot & \cdot & \gamma_{M}^{N-1} \end{array}\right]\left[\begin{array}{c} R_{1}\\ R_{2}\\ \cdot \\ \cdot \\ R_{M}\; \end{array}\right]=\left[\begin{array}{c} f_{1}\\ f_{2}\\ \cdot \\ \cdot \\ f_{N-1}\; \end{array}\right]$$

With the information of $R_{i}$ and $s_{i}$, it is possible to generate the summary in Equation (2), thus making a reliable reconstruction of the original signal [27].

### 2.2. Time Reversal Method

Time reversal method (TR) is an effective tool to solve problems related to the characterization of signals in processes of the location of targets in an inhomogeneous medium. Consider a wave propagating with an average velocity c(r) through a determined non-linear medium, with a characteristic Equation (p(r,t)) which satisfies [7]:

(13) $$\Delta p=\frac{1}{c^{2}\left(r\right)}\frac{\delta^{2}}{\delta t^{2}}p=0$$

where *t* is the time and *r* is related to the space coordinated inside the medium. Consider that Equation (13) has a second-order time derived, implying the time-reversal invariant property if the medium has lossless propagation. Thus, once p(r,t) is defined as a solution for the equation, it is possible to determine that p(r,−t) also represents a solution [7]. If we consider the propagation of a plane wave through two different media M1 and M2, it is not possible to assure the time-reversal property in the solution of the wave equation. Now, if the incident wave travels from M1 to M2, we can detect a reflected wave R in M1 and a transmitted wave T in M2.

We can now define the reflection coefficient $R^{\prime }$ and the transmission coefficient $T^{\prime }$ related to the wave coming from the waves. Now, considering the superposition principle in M1 with $R^{2}+TT^{\prime }$ and in M2 with $RT+TR^{2}$, it is possible to verify that [29]:

(14) $$R^{2}+TT^{\prime }=1$$

(15) $$R+R^{\prime }=0$$

Relations in Equations (14) and (15) allow us to extrapolate the time-reversal property, previously mentioned in the propagation of planar waves through two different media. Thus, it is possible to implement TR in non-homogeneous media.

**Figure 1:**
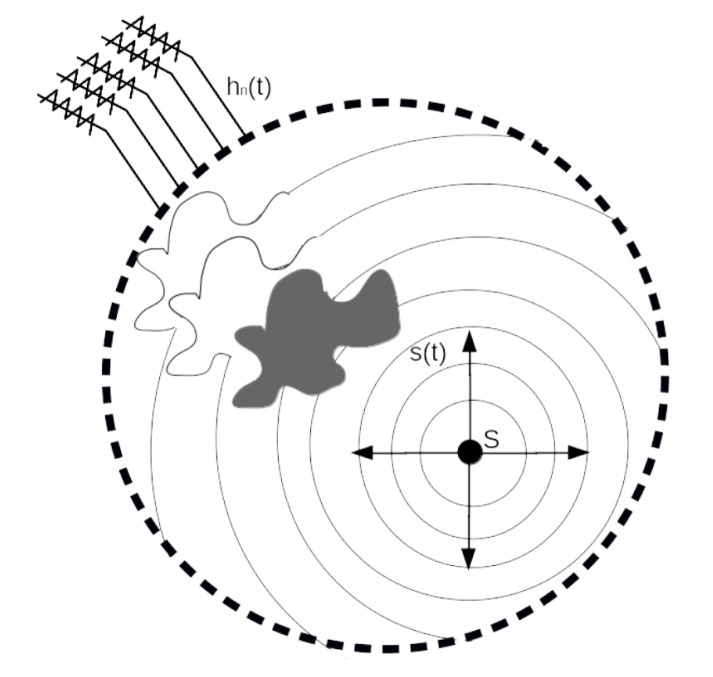
Transmission of the incident wave from source to sensors.

Consider the case of Figure 1, where we have a set of a certain number (ideally infinite) of sensors around a determined source (S). S produces a signal s(t), which is propagated through an inhomogeneous medium. Our principal goal is to determine the location of S, estimating a solution p(r,−t) for the wave equation. When the spherical wavefront is propagated, it is deformed by the non-homogeneities that are in the medium. After that, the signal $h_{n}\left(t\right)$ arrives at the *n*-th sensor and is composed by

(16) $$h_{n}\left(t\right)=f\left(t-t_{n}\right)-u\left(t\right)+noise\left(t\right)$$

Here, $t_{n}$ is the elapsed time between the start of the signal and the moment when it arrives at the sensor. u(t) are deformations produced by inhomogeneities, and noise(t) is the noise present in the medium. The location process and the estimation of u(t) and noise(t) are impossible with the information of a single sensor. Nevertheless, we can forget this problem if we use all $h_{n}\left(t\right)$ signals. Thus, if we have more sensors, we can obtain a better estimate of the position.

Let us define $h_{n}\left(T-t\right)$ as the signal at the *n*-th sensor, with a start in t=0 and a duration of T seconds. T must be a value that ensures that all sensors can record, at least, the beginning of the S signal. Now, we can define $h_{n}\left(T-t\right)=h_{n}\left(-t\right)$ as the time-inverted signal at the n-th sensor. If we backpropagate $h_{n}\left(-t\right)$ from each sensor through the medium, we can find the position of S. Remember that it is possible because we defined that p(r,−t) is also a solution to the wave equation.

**Figure 2:**
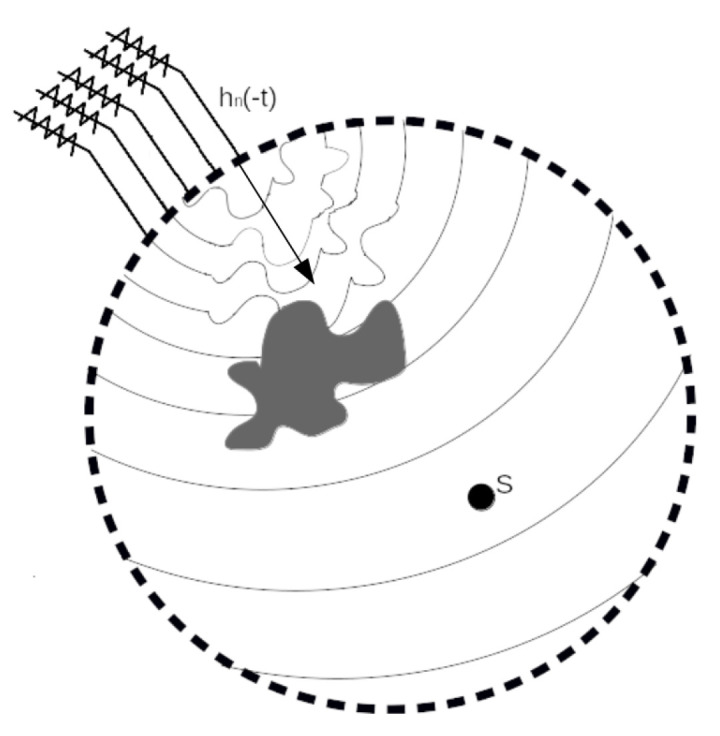
Backpropagation of $h_{n}\left(t\right)$ signals.

Figure 2 shows the second step in the TR process. Here, we take $h_{n}\left(-t\right)$ and send it through the medium until it arrives at the S position. Now, u(−t) is included in the backpropagation process of $h_{n}\left(-t\right)$. Its effect is canceled as the signal spreads through the inhomogeneities. Thus, when $h_{n}\left(-t\right)$ arrives at S, we can estimate the position. Note that if we have a greater number of sensors, we can better estimate the position because this reduces the deformations from all angles.

## 3. Proposed Method

The general purpose of our work is the treatment of underwater acoustic signals coming from ship engines to calculate an accurate location of the vehicle. This process is carried out using only passive sensors, and it is considering a previous data reduction process that optimizes the bandwidth of a wireless communication channel.

For the latter, we have the final purpose of installing four buoys with hydrophones that collect the signal in the middle of the water. Each buoy will send a reduced data set using a wireless communication channel. MPM is the technique used to reduce the amount of information from the signals in the sensors. After that, the signals will be placed in a central node, and the localization process will be carried out using the TR method.

The general idea is to carry out the backpropagation of the signals in a simulated way to reach an accurate identification without using active sensors. However, in this work, the initial propagation is also presented in a simulated way to demonstrate that the signals previously treated with MPM can be correctly located.

### 3.1. Matrix Pencil Method

#### 3.1.1. Signal Test

**Figure 3:**
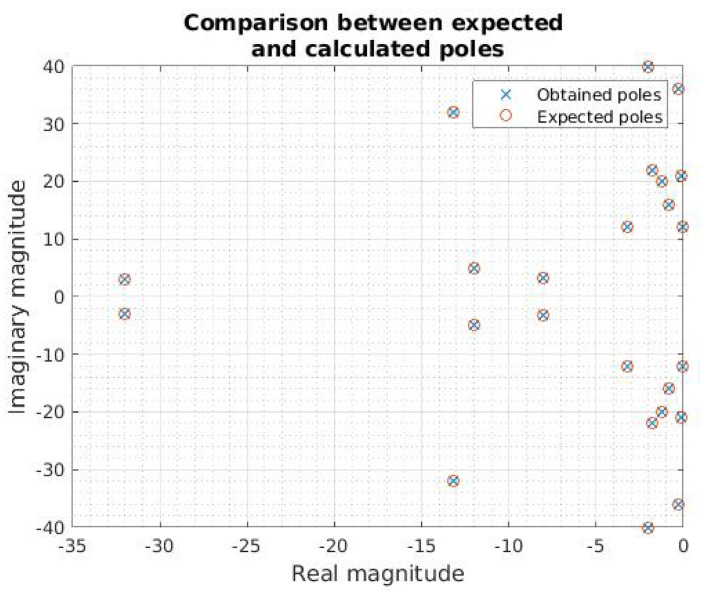
Comparison between expected poles and obtained poles in the signal test.

To perform the first tests concerning MPM, we digitally created signals. The purpose of this process was to test the operation and efficiency of the method in signals with known singularities. Capitalizing on the information above and considering the theory described by Equations (2) and (3), we proceeded to the digital generation of a signal with 24 pre-established singularities. After that, we processed it with MPM and contrasted the information with the expected poles. Figure 3 shows the comparison between the poles previously established in the signal generation and those calculated after applying MPM.

Figure 3 shows that after the MPM process, it is possible to find the poles related to a signal with known singularities. However, the comparison between the original and the rebuilt signals should not be made graphically, but using more precise mathematical methods. For this purpose, we used the “coefficient of determination $R^{2}$”, defined considering the linear regression model as [30]

(17) $$y_{i}=X_{i}\beta +\epsilon_{i},i=1,2,\ldots ,n$$

where $y_{i}$ represents the *i*-th element of the *Y* signal with a size of *n*, $X_{i}$ is the *i*-th row of the design matrix $X_{nxp}$, and β represents a column vector of *p* positions related to the unknown regression coefficients, and $\epsilon_{i}$ is defined as the Gaussian normal distribution ($N(0,\sigma^{2})$). Thus, $R^{2}$ is defined as

(18) $$R^{2}=1-\frac{SSE(X)}{SSE(1_{n})}$$

where:

(19) $$SSE\left(X\right)=\sum_{i=1}^{n}{\left(y_{i}-X_{i}\hat{\beta }\right)}^{2}$$

(20) $$SSE\left(1_{n}\right)=\sum_{i=1}^{n}{\left(y_{i}-\bar{y}\right)}^{2}$$

In our specific case, correlation $R^{2}$ between the original and the rebuild signal can be expressed as a percentage derived to $0\leq R^{2}\leq 1$. Thus, the comparison in Figure 3, where all the original poles were rebuilt, indicates a $R^{2}$ of 100%.

#### 3.1.2. Definition of M

Mckenna (2012) in [31] states that the principal components of the signal of motors of vessels are below 1 kHz. Then, according to the Nyquist theory, the minimum sampling rate should be 2 kHz [32]. In our case, we used a bulk carrier signal with a sampling rate of 2.2 kHz. Mckenna (2012) also indicates that the fundamental components of the ships used in their work are predominantly above 40 Hz [31]. Thus, a signal with a duration of one second contains the general information related to the ship, time that we use for our test. This signal is part of a private database belonging to the Colombian army.

**Figure 4:**
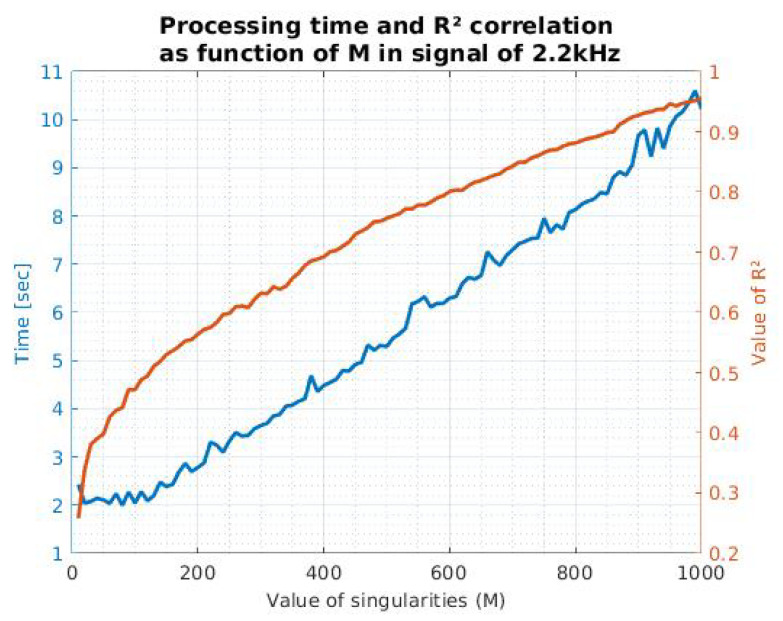
Processing time and correlation coefficient as a function of M, in a bulk carrier ship signal with a sample rate of 2.2 kHz.

In order to define the most appropriate value of M, we used the process proposed in Equation (7). We discovered that for the signal mentioned above, with p=2, the M value could be above 1600. Likewise, we found that in general, this value depends on the characteristics and nature of the treated signals. Therefore, we decided to evaluate $R^{2}$ and the processing time as functions of M, and we obtained the information shown in Figure 4. Computational load corresponds to the processing time in an AMD Ryzen 7 3700U with Radeon Vega Mobile Gfx of 64 bits.

**Figure 5:**
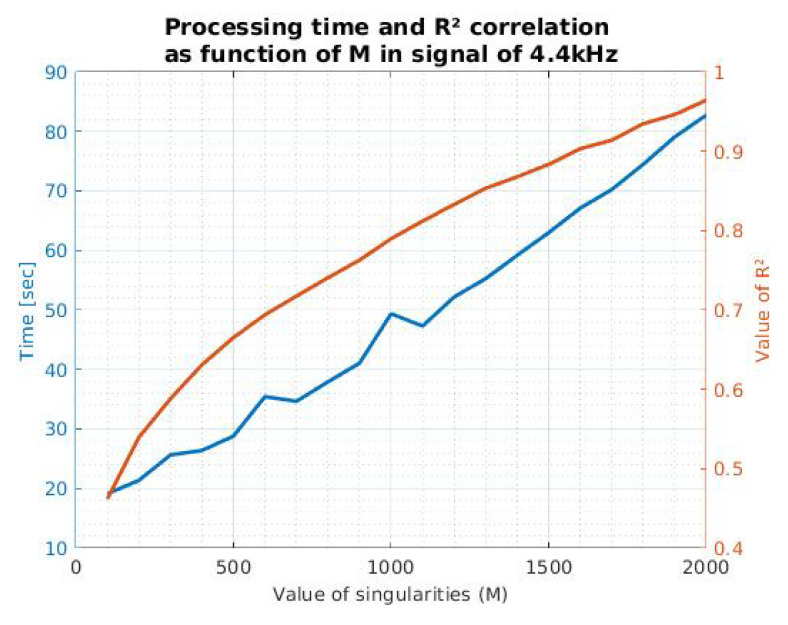
Processing time and correlation coefficient as a function of M, in a bulk carrier ship signal with a sample rate of 4.4 kHz.

In order to define how the relation of the parameters treated in Figure 4 changes as a function of the number of points, we proceeded to perform the comparisons using the same signal, but with a different sample rate (4.4 kHz). Figure 5 shows how these parameters vary as a function of M for this particular case.

It is possible to mention that in the case of Figure 5, to achieve a correlation above 90%, at least 1600 poles are required, with an estimated processing time of 67.10 s. In contrast, in the signal of Figure 4, to obtain these same reconstruction parameters, 860 singularities are required, with a processing time of 8.8 s. This information indicates that M and time processing are directly proportional to the number of points corresponding to the signal of interest and thus could affect the method efficiency.

Similarly, we found that the relationship between M and $R^{2}$ depends on the parameters of the medium and the measurement instruments. For this reason, we recommend that an analysis such as those presented in Figure 4 and Figure 5 could be useful to define the propitious value of M. Moreover, this definition could help with the characterization of the medium, which can be carried out with recordings without boats. This process could eliminate the poles calculated with MPM belonging to the background noise to leave only the signal of interest. Furthermore, this could broaden the scope of the recording.

Considering the information mentioned above, it is clear that the definition of M can become a complicated process for the implementation of the method in natural signals. Is it necessary to perform a correlation and processing analysis, as shown in Figure 4 and Figure 5. Consequently, this information must be collected by the final instruments and in the required environment. For our case, we opted for a minimum correlation of 90%, which means a value of M of at least 860.

### 3.2. Time Reversal Method

For the TR implementation, based on the information in Section 2.2, the simulation of the propagation and backpropagation was carried out. As a working grid, we defined a 100 × 100 matrix for a delimited area of 1 km${}^{2}$, with a distance between the rows and columns of 10 m. The precision of the method and the computational load are directly proportional to the number of points in the grid. Capitalizing on the size of the targets we want to track, we estimated that the distance in the grid, every 10 m is sufficient to give a good approach to the location.

Our proposed localization process calculates the estimated points with the highest amount of energy throughout the work area after the backpropagation process. The error in this estimation depends on the distance between the points of the grid (10 m in our case). Then, TR estimates the position of the target within a delimited area to around a point in the grid, not in an exact location. The same occurs with the sensors, whose position can be within the established area between two grid points. In our case, this position can vary by more or less than 5 m on the X and Y axes.

Its location may change as a function of time due to environmental effects. Because of this, the GPS monitoring of hydrophones can adjust the changes well to perform TR with the pertinent positional considerations. Wang (2021) addresses some methods adjusting the changes of position on the buoys [33].

Once the work area was defined, we focused on the specifications of the parameters. We determined the location and number of sensors, such as the initial location of the source. Specifically for our project, we carried out the process using a maximum of four sensors. Thus, in our preliminary simulation, we used an array of hydrophones with a symmetrical rhombus shape.

Once the simulation parameters were determined, we proceeded to propagate the signal generated by the source. For this, we proliferated a real signal from a landing boat vehicle, of 3 s of duration and a sample rate of 4.4 khz, along the grid until it reached each of the sensors. For the propagation of the signal, we must take into account the inherent delay in the speed of sound (for seawater, approximately 1500 m/s) and the attenuation as a function of the distance of the wave. For this fact, we used the solution to the plane wave equation:

(21) $$p\left(r,t\right)=Ae^{-j(\omega t-kr)}$$

where *A* is defined by the magnitude in f(t) and its attenuation as a function of distance, ω is the angular frequency, *k* the wavenumber and *r* distance.

At this point, we are simulating the propagation of the landing boat signal, collecting information in each sensor. In practice, this process would be experimental, not like backpropagation, which must be simulated to guarantee the calculation of the location without using active sensors. Once the signal information is available in each of the sensors, regardless of whether the signal collection is simulated or experimental, $f_{n}\left(-t\right)$ is defined and the signals are backpropagated, as a summation in each of the grid points following Equation (21).

To estimate the location of the source, we calculate the total power at each of the grid points ($P_{\left(n,m\right)}$), following the Equation (22). Where *m* and *n* are in point position, *T*, the total time of f(t) and $g_{n,m}$ is the sum of the backpropagated signals at point n,m:

(22) $$P_{\left(n,m\right)}=\int_{0}^{T}{\left(f_{n,m}\left(t\right)\right)}^{2}dt$$

**Figure 6:**
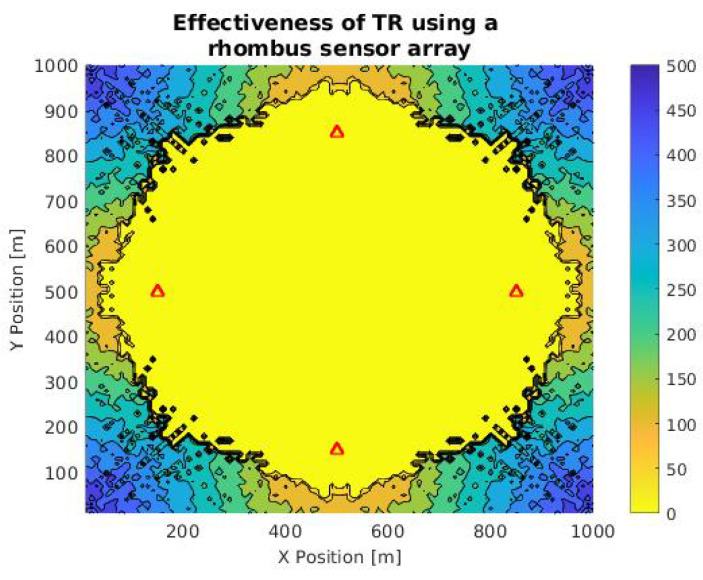
Contour plot of TR calculation accuracy in a rhombus-shaped array in an area of 1 km${}^{2}$ for a landing ship signal. Red triangles are sensor positions and the color scale is a function of the difference between the expected and calculated location.

We decided to verify the method’s effectiveness in simulation by varying the source’s location throughout all points in the grid and estimating the difference between the location proposed and the location calculated using TR. Figure 6 shows the contour plot in a case where sensors have a rhombus-shaped distribution. The location of sensors is represented as the shaded triangles and the difference between S and the calculated position is shown as the contour distribution, where the function of the distance between those points in meters is represented with a color scale: yellow represents the lowest error calculation (0 m) and dark blue is the highest value in the simulation process (500 m).

The average distance between the position of the source and the simulated value is 0 m inside the rhombus shadow generated by the sensors array. Thus, inside this area, the position calculation of a target is carried out correctly using the TR method. On the other hand, we note that outside of the rhombus mentioned above, the accuracy decreases. The error value increases as a function of the distance. The highest error values are presented in the corners of the grid (above 485 m). Likewise, we should consider that the range and the maximum distance between the sensors will depend on environmental factors such as propagation speed, the attenuation coefficient, and ambient noise. Then, it indicates that the characterization of the medium is a fundamental axis for the implementation of the method in real environments.

## 4. Method Validation

### 4.1. Data Reduction Produced by MPM

Our final objective will be recording the signals of boats in the middle of the ocean, which is why a wireless communication channel is needed. This requirement implies the highest possible reduction in information in the communication process. Fortunately, MPM facilitates this procedure. Taking as an example the signal of a bulk carrier related to Figure 4, we have a 2200 positions vector, corresponding to 1 s with a sampling rate of 2.2 kHz. Each position matches a double number (8 bytes), and the array has a size of 141 Kbits [34].

After using the MPM, we have M complex double numbers (16 bytes). Therefore, it implies that the total number of bits (Tnb) is:

(23) Tnb=128M

We notice that, considering Fourier theory, in Figure 3, each singularity with an imaginary positive part has a corresponding complex conjugate. This information is redundant and could be omitted to reduce the number described by Equation (23) on the communication process. Therefore, the Tnb value is reduced to:

(24) Tnb=64M

Data reduction is inversely proportional to the correlation coefficient, so these parameters need to be defined depending on the application and the medium restrictions. In the case of our bulk carrier signal, we can talk about the percentages described in Table 1. Thus, to ensure a minimum reconstruction of 90%, a value of M=680 is necessary, obtaining a data reduction of 69.13%.

### 4.2. Location Simulation Process

#### 4.2.1. Simulation Analysis Derived by the Change in the Distribution of the Sensors

Considering the distribution observed in the simulation of Figure 6, related to a landing boat, we opted for the simulation of a time reversal mirror (TRM), which consists of a linear arrangement of sensors that allows the TR calculation in an orthogonal direction from the array [29], using the same signal from Figure 6. The purpose of this simulation was to compare its efficiency, obtained using the rhombus arrangement.

**Figure 7:**
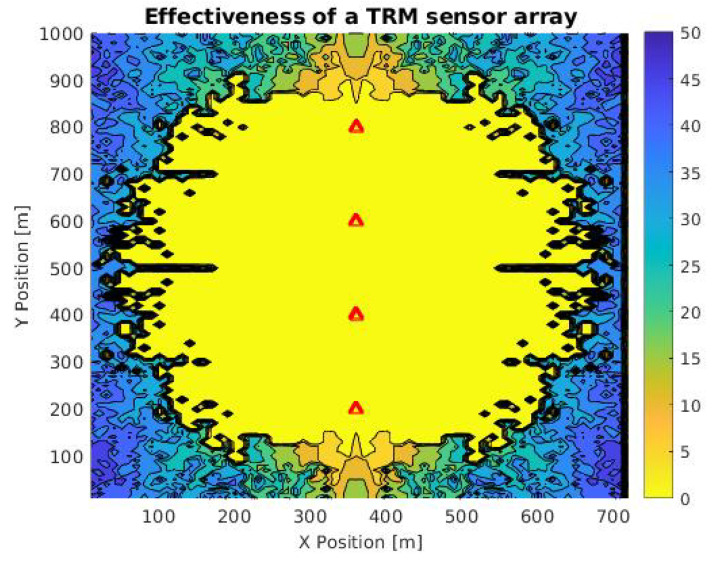
Contour plot of the accuracy in TRM array in an area of 0.72 km${}^{2}$ for a landing ship signal. Red triangles are sensor positions and the color scale is a function of the difference between expected and calculated location.

Figure 7 shows the contour plot of the TRM arrangement on a 720 × 1000 m grid with a point spacing of 10 m. In addition, the formatting guidelines (color and marking) are consistent with the simulation of Figure 6. Here, it is possible to identify an ellipse related to the points where the calculation error is equal to 0 m. The ellipse center is in the midpoint of the four sensors and the maximum distance, which is orthogonal to the arrangement, is approximately 450 m.

Note that the shadow of the ellipse is around the sensors, which indicates that the array can calculate the target location symmetrically to the right and left. Similarly, the simulation with this arrangement allows calculating the maximum range of the sensors (approximately 450 m). This value is specific for this case, and it depends on specific environmental conditions. We find that under the same signal and environment parameters, the rhombus-shaped arrangement has a total number of points with zero error of 6020, while in the case of the TRM, it is 4007. These values will also depend on the distance between the sensors and the quality of the signal.

Note that the changes of frequency in the observer, produced by the Doppler effect, are not taken into account in our simulations. The latter is because the presented cases refer to a controlled environment with a fixed and immobile objective. However, this phenomenon must be considered in future steps of the work to determine how changes in the frequency and magnitude in the observer signal affect the total power levels in the grid and the subsequent localization process.

#### 4.2.2. Simulation Analysis Derived by the Change in Signal of Interest

**Figure 8:**
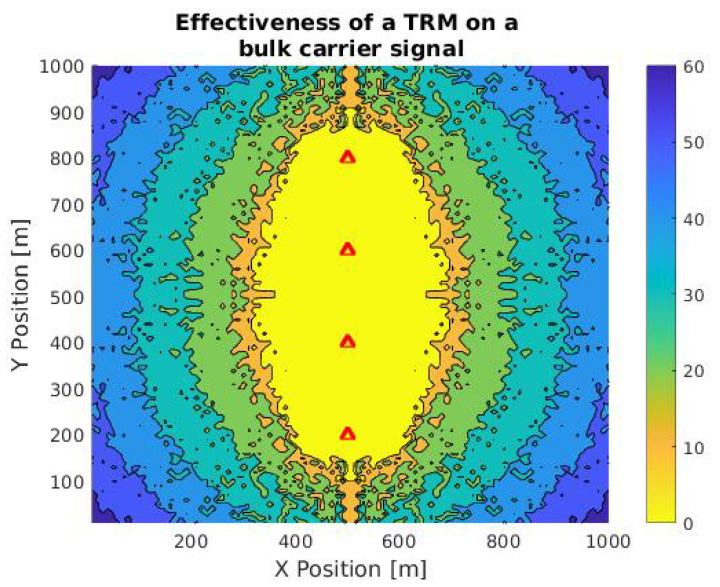
Contour plot of TR calculation accuracy in a TRM in an area of 1 km${}^{2}$ to the Bulk carrier signal. Red triangles are sensor positions and the color scale is a function of the difference between expected and calculated location.

Similar to the previous process, a TRM simulation was performed using the bulk carrier signal related to Figure 4 and Figure 5. The differences between this signal and the one from the landing boat, in Figure 6 and Figure 7, are the sampling rate, time, signal-to-noise ratio, and its total power. In the case of the landing boat, we have a signal of 3 s of duration, with a sampling rate of 4.4 kHz, total noise of −6.1381 dB, and a harmonic distortion power of −8.2218 dB. On the other hand, the bulk carrier signal has 1 s with a sampling rate of 2.2 kHz, the noise of −9.6888 dB, and harmonic distortion power of −51.2480 dB. This is a good indicator to evaluate the difference in the performance of the method with the signal variations caused by the ships and measurement conditions. Figure 8 shows the efficiency analysis in the case of the bulk carrier signal in a grid under the same conditions as Figure 6.

Note that the area delimited by the yellow color, which determines the positions with zero error, is smaller than that presented in Figure 8. The total range orthogonal to the sensors also drops considerably, from approximately 500 to 350 m. Thus, the total amounts of energy and noise of the signal have a key role in the precision of the method. This must be carefully considered in real implementations.

#### 4.2.3. Simulation Analysis of the Signals Treated with MPM

Once the MPM process is implemented in the acoustic signals for the compression process and their information is sent through wireless channels, we can begin with the location process using TR. Our goal is to prove that it is possible to estimate the location of a target using only passive sensors in an inhomogeneous medium. Nevertheless, due to logistical difficulties, real experimentation is a task for the future.

**Figure 9:**
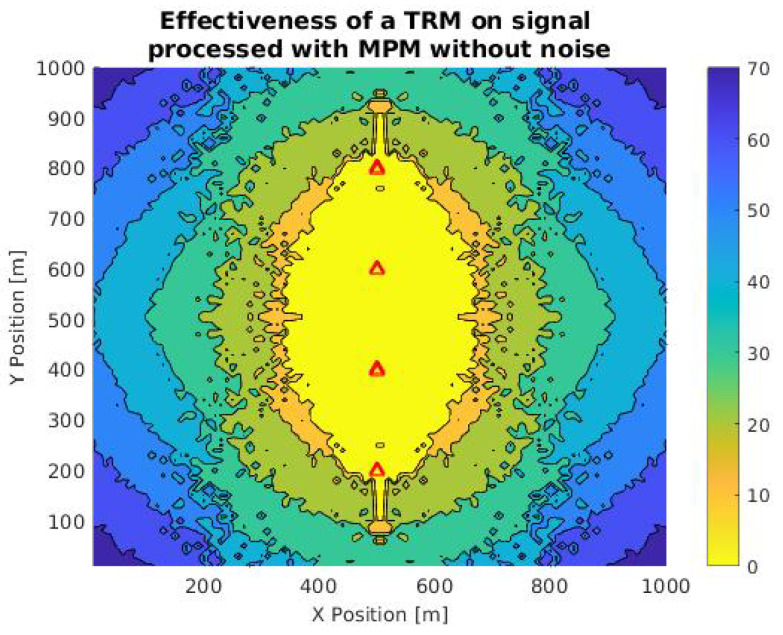
Contour plot of the accuracy in the TRM array in an area of 1 km${}^{2}$ to a bulk carrier signal previously treated with MPM (*M* = 860). Red triangles are the positions of the sensor and the color scale is a function of the difference between the expected and calculated location.

For determining the difference between the efficiency of TR in pure signals and signals previously treated with MPM, we decided to implement the simulation of Figure 9 following the same parameters of the simulation of Figure 8 (size, environmental conditions, position of sensors). This simulation was performed by using the information provided by MPM after the reconstruction of the signal, with the parameters described in Section 3.1.2 (*M* = 860 and $R^{2}=90\%$).

Note that in comparison to the original simulation; the number of points with an error equal to 0 is slightly smaller. Specifically, for the case of the pure signal, there are 2016 points in which the error is null, while in the case of the reconstructed signal, there are 1552 points. This represents a change in the efficiency of 23%, which can be considered low according to the reduction in information in the communication process.

#### 4.2.4. Simulation Analysis of Signals Treated with MPM, Including Noise Levels

**Figure 10:**
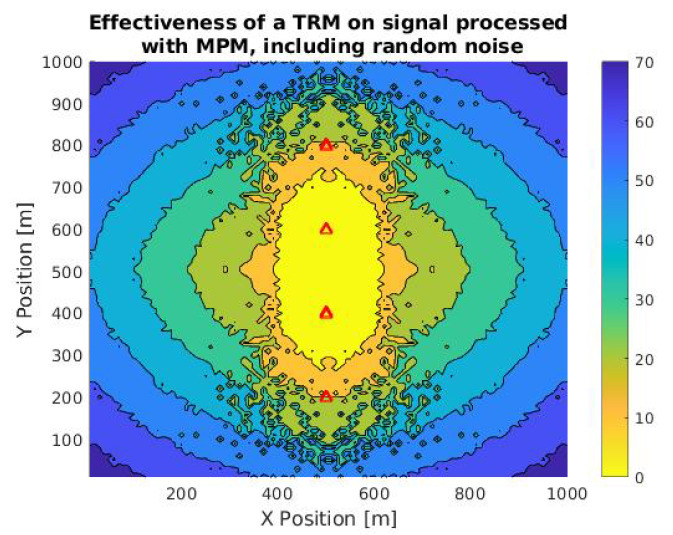
Contour plot of TR calculation accuracy in a TRM in an area of 1 km${}^{2}$ to a bulk carrier signal previously treated with MPM (*M* = 860), and adding random noise in the propagation of the waves. Red triangles are the positions of the sensor and the color scale is a function of the difference between the expected and calculated location.

We need information about how the noise presented in the environment can also affect the localization process. For this reason, after adding random noise of −48 dB in the propagation and backpropagation threads, we obtained the simulation of Figure 10.

In this simulation, we obtained 732 points of null error, in contrast to the points of the signal without noise (1552). This difference confirms that the method efficiency is inversely proportional to the background noise and the signal-to-noise ratio. One way to improve this problem is to estimate the fundamental components related to the noise of the environment, using MPM. This characterization could reduce the noise levels in the reconstructed signal and improve the effectiveness and scope of the method. Although this can compromise the correlation between the original signal and the reconstructed one, as only ambient noise would be eliminated, so the information from the ship signal would be cleaner.

## 5. Conclusions

We demonstrated a method based on MP for the compression of acoustic signals captured by hydrophones. Simulated results show data compression percentages above 60%, with a correlation coefficient of 90%. The subtraction of the estimated noise levels of the environment helps to improve the calculation of specific singularities in the acquired signal.

The main problem in MPM implementation is the definition of the M parameter, which strictly depends on the nature of the signal, the bandwidth and computational load requirements. We found that the processing time is proportional to the signal size, which could affect the final application. Therefore, a requirement analysis must be performed on the signal to use a vector that is as small as possible.

We showed the implementation of TR in signals compressed by MPM in two different types of ships using two arrays. Furthermore, we also analyzed how ambient noise and quality in the reconstruction interfere with the localization process. Specifically, we find that the scope of the location depends on the power of the signal of interest and the signal-to-noise relation. Likewise, we found that the implementation of TR in signals treated with MPM (correlation of 90%) provides a difference in the performance of 23% with respect to the original signals. The accuracy of the localization process could be improved by characterizing the representative poles of the noise in the environment so as not to include them in the calculations.

The principal difficulty of our method is the identification of the characteristics of the medium and the signal. For the identification process, the signals must be backpropagated in a simulated environment. Due to this fact, it is necessary to experimentally estimate the average propagation speed and the average attenuation. Likewise, we recommend carrying out a previous analysis related to the noise present on the medium and the relation of the signal with the correlation considering the M value.

## Figures and Tables

**Table 1:** Information about the compression of a bulk carrier signal with rate sampling of 2.2 kHz.

*M*	Compression Percentage (%)	Correlation Coefficient ($R^{2}$)
10	99.09	0.25
50	97.72	0.39
80	96.36	0.441
100	95.45	0.4715
200	90.909	0.5638
300	86.363	0.6318
400	81.81	0.6922
500	77.305	0.756
600	72.727	0.803
700	68.182	0.842
800	63.687	0.8812
900	59.14	0.9312
1000	54.54	0.955

## Data Availability

Not applicable.
